# Development of rotational intraperitoneal pressurized aerosol chemotherapy to enhance drug delivery into the peritoneum

**DOI:** 10.1080/10717544.2021.1937382

**Published:** 2021-06-12

**Authors:** Soo Jin Park, Eun Ji Lee, Hee Su Lee, Junsik Kim, Sunwoo Park, Jiyeon Ham, Jaehee Mun, Haerin Paik, Hyunji Lim, Aeran Seol, Ga Won Yim, Seung-Hyuk Shim, Beong-Cheol Kang, Suk Joon Chang, Whasun Lim, Gwonhwa Song, Jae-Weon Kim, Nara Lee, Ji Won Park, Jung Chan Lee, Hee Seung Kim, On behalf of the KoRIA* trial group

**Affiliations:** a Department of Obstetrics and Gynecology, Seoul National University Hospital, Seoul, Republic of Korea; b Interdisciplinary Program in Bioengineering, Seoul National University Graduate School, Seoul, Republic of Korea; c Institute of Animal Molecular Biotechnology and Department of Biotechnology, College of Life Sciences and Biotechnology, Korea University, Seoul, Republic of Korea; d Department of Obstetrics and Gynecology, Dongguk University Ilsan Hospital, Goyang, Korea; e Department of Obstetrics and Gynecology, Research Institute of Medical Science, Konkuk University School of Medicine, Seoul, Republic of Korea; f Department of Experimental Animal Research, Biomedical Research Institute, Seoul National University Hospital, Seoul, Republic of Korea; g Department of Obstetrics and Gynecology, Ajou University School of Medicine, Suwon, Republic of Korea; h Department of Food and Nutrition, Kookmin University, Seoul, Republic of Korea; i Department of Obstetrics & Gynecology, CHA Gangnam Medical Center, CHA University, Seoul, Republic of Korea; j Department of Surgery, Seoul National University College of Medicine, Seoul, Republic of Korea; k Department of Biomedical Engineering, Seoul National University College of Medicine, Seoul, Republic of Korea; l Institute of Medical and Biological Engineering, Medical Research Center, Seoul National University, Seoul, South Korea

**Keywords:** Intraperitoneal chemotherapy, doxorubicin, pharmacokinetics, drug delivery, peritoneal metastasis

## Abstract

This study aims to evaluate the drug distribution, tissue concentrations, penetration depth, pharmacokinetic properties, and toxicities after rotational intraperitoneal pressurized aerosol chemotherapy (RIPAC) in pigs. Because relevant medical devices have not been introduced, we developed our prototype of pressurized intraperitoneal aerosol chemotherapy (PIPAC) and RIPAC by adding a conical pendulum motion device for rotating the nozzle. RIPAC and PIPAC were conducted using 150 ml of 1% methylene blue to evaluate the drug distribution and 3.5 mg of doxorubicin in 50 ml of 0.9% NaCl to evaluate the tissue concentrations and penetration depth, pharmacokinetic properties, and toxicities. All agents were sprayed as aerosols via the nozzle, DreamPen^®^ (Dalim Biotech, Gangwon, South Korea), with a velocity of 5 km/h at a flow rate of 30 ml/min under a pressure of 7 bars, and capnoperitoneum of 12 mmHg was maintained for 30 min. As a result, RIPAC showed a wider distribution and stronger intensity than PIPAC. Compared with PIPAC, RIPAC demonstrated high values of the tissue concentration in the central, right upper, epigastrium, left upper, left lower, right lower, and right flank regions (median, 375.5–2124.9 vs. 161.7–1240 ng/ml; *p* ≤ .05), and higher values of the depth of concentrated diffusion and depth of maximal diffusion (median, 232.5–392.7 vs. 116.9–240.1 μm; 291.2–551.2 vs. 250.5–362.4 μm; *p* ≤ .05) in all regions except for bowels. In RIPAC, the pharmacokinetic properties reflected hemodynamic changes during capnoperitoneum, and there were no related toxicities. Conclusively, RIPAC may have the potential to enhance drug delivery into the peritoneum compared to PIPAC.

## 1. Introduction

Peritoneal metastasis (PM) occurs in up to 60% of advanced or recurrent diseases of solid tumors (Heintz et al., 2006; Quere et al., 2015; Yarema et al., 2020), which leads to a poor expected median survival of fewer than 20 months despite various types of intravenous chemotherapy (Robella et al., 2016). Specifically, intravenous chemotherapy shows little effect on improving the prognosis of patients with PM because of the insufficient blood supply to the peritoneal surface with low penetration into tumors, thereby preventing eradication (Thadi et al., 2018). As an alternative, intraperitoneal chemotherapy has been attempted to treat PM of solid tumors by the direct contact of chemotherapeutic agents to the tumors without reliance on the blood supply for overcoming the limitations of intravenous chemotherapy

In a specific way, early postoperative intraperitoneal chemotherapy (EPIC) and hyperthermic intraperitoneal chemotherapy (HIPEC) have been used immediately after maximal cytoreduction for treating PM of solid tumors. Before applying EPIC and HIPEC, maximal cytoreduction leaving residual tumors of less than 2.5 mm is important for effective drug delivery even with limited penetrations of 1–3 mm during intraperitoneal chemotherapy (Witkamp et al., 2001). Moreover, hyperthermia can increase the penetration of chemotherapeutic agents and enhance drug sensitivity by impairing DNA repair, inducing apoptosis, and promoting the denaturation of proteins (Spratt et al., 1980; van de Vaart et al., 1998). However, catheter-related complications and renal or hepatic toxicity reduce the treatment cycles to achieve an insufficient effect, and their effectiveness has not been demonstrated in some types of solid tumors and recurrent diseases (Ishigami et al., 2018; Kim et al., 2019; Klaver et al., 2019).

Pressurized intraperitoneal aerosol chemotherapy (PIPAC) has been introduced as palliative therapy for treating PM in recurrent diseases (Solass et al., 2014; Grass et al., 2017). It delivers chemotherapeutic agents as aerosols with a median diameter of 25 μm under a pressure of 200 psi made by a high-pressure injector (Solaß et al., 2012). In particular, PIPAC has the advantages that only 10% of the dose of the chemotherapeutic agents used in intravenous chemotherapy is sprayed diffusely throughout the peritoneal cavity with fewer toxicities, and the tissue concentration after PIPAC is maintained up to 200 times that achieved after intravenous chemotherapy by interrupting the venous circulation by capnoperitoneum of 12 mmHg made using a laparoscopic system, thereby suppressing systemic excretion of the agents (Blanco et al., 2013; Robella et al., 2016).

Nevertheless, restricted use due to dissemination in only some European countries and the uneven distribution and penetration in various regions of the peritoneal cavity act as disadvantages of PIPAC (De Andrade et al., 2019). To overcome these limitations of PIPAC, the KoRIA (Korean Rotational Intraperitoneal pressurized Aerosol chemotherapy) trial group developed rotational intraperitoneal pressurized aerosol chemotherapy (RIPAC) by adding a remote-controlled device for rotating the PIPAC nozzle (Mun et al., 2021), and this study showed preclinical evidence that RIPAC may improve drug delivery compared to PIPAC with fewer toxicities in pigs.

## 2. Materials and methods

### 2.1. Rotational intraperitoneal pressurized aerosol chemotherapy system

For delivering doxorubicin as aerosols, we used our prototype for PIPAC, which sprayed approximately 30-µm sized droplets through the nozzle, DreamPen^®^ (Dalim Biotech, Gangwon, South Korea), with a velocity of 5 km/h at a flow rate of 30 ml/min under a pressure of 7 bars equivalent to about 100 psi (Lee et al., 2020). The mean diameter of the sprayed region was 18.5 cm, and the penetration depth ranged from 360 to 520 µm, comparable to previous studies using the microinjection pump (Capnopen^®^; Capnomed, Villingendorf, Germany) (Khosrawipour et al., 2016b; Gohler et al., 2017).

**Figure 1.**
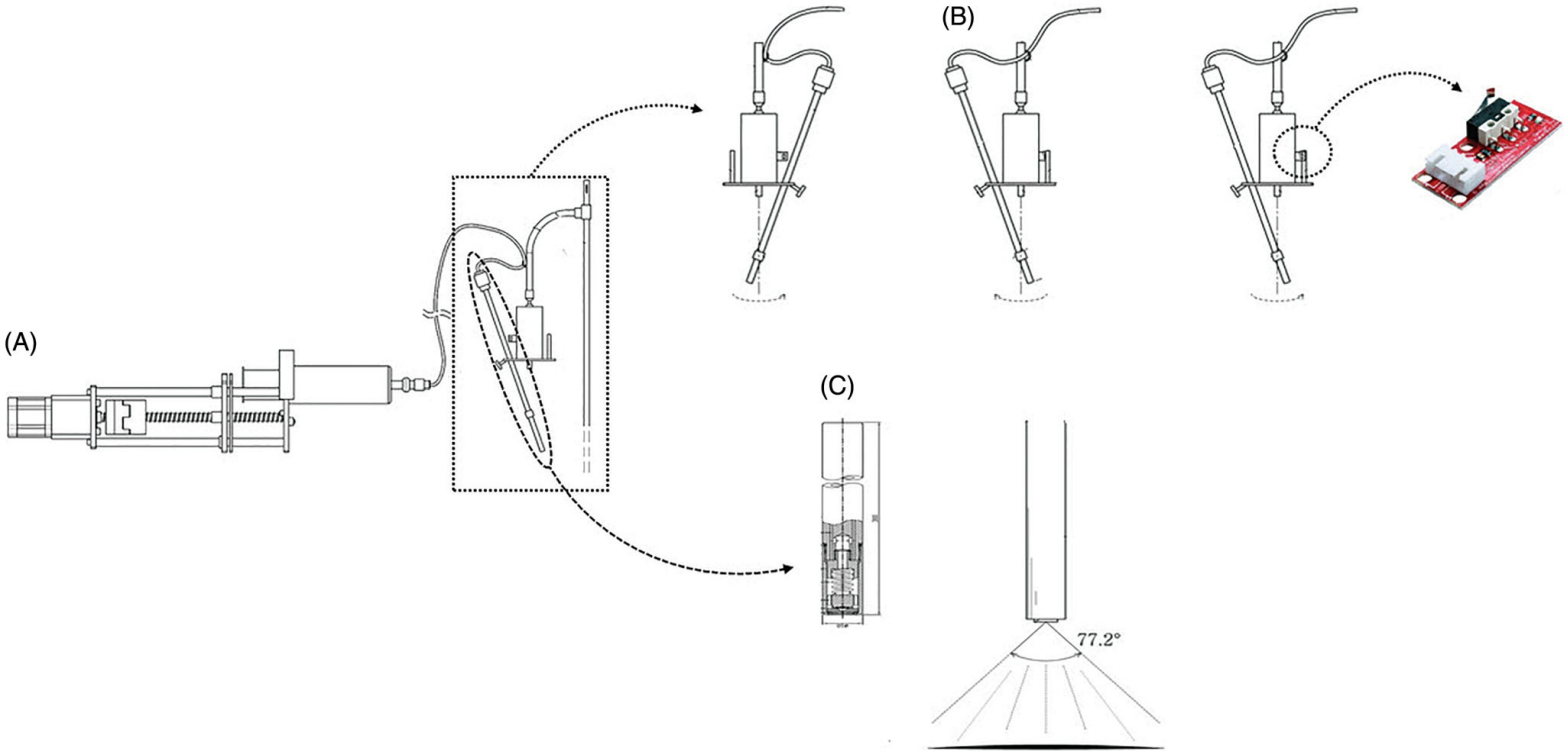
Schematic diagram of rotational intraperitoneal pressurized aerosol chemotherapy (RIPAC). (A) A high-pressure injector to generate a pressure of 7 bars (=101 psi), (B) the conical pendulum motion device for rotating the nozzle during RIPAC, and (C) the spraying angle of 77.2 degrees.

For RIPAC, we added a remote-controlled conical pendulum motion device to our prototype for PIPAC and rotated the nozzle to improve drug delivery. The conical pendulum motion device was composed of a DC motor (12 V/1.5A, GM35A-3323, Motorbank, Seoul, South Korea), a 3-D printed rotational stick, two end-stops (PCB-mounted End-stop switch, RepRap, England), and an Arduino Uno. We inserted the nozzle in a 3-D printed rotational stick and locked it with a screw. The angle between the nozzle and the vertical line was determined at 30 degrees by considering a spraying angle of 77.2 degrees. The rotational stick could not rotate in the same direction because the tube line connected between the nozzle and the syringe pump became tangled. Thus, the rotational stick moved clockwise, and when the rotating stick contacted the sensor of the rotating path, it moved counterclockwise to maintain repetitive rotation (Figure 1) (Mun et al., 2021).

### 2.2. Reagents

We purchased 1% methylene blue and doxorubicin from Sigma-Aldrich (St. Louis, MO, USA) for intraperitoneal chemotherapy. For analyzing the serum and tissue concentrations of doxorubicin, we purchased acetonitrile and methanol from Fisher Scientific (Waltham, MA, USA) and formic acid, acetic acid, and ammonium acetate from Sigma-Aldrich. We bought 1.5 µg/ml 4′,6-diamidino-2-phenylindole (DAPI) from Sigma-Aldrich to evaluate the penetration depth of doxorubicin.

### 2.3. Preparation

This study was approved by the Institutional Animal Care and Use Committee (IACUC) of Seoul National University Hospital before study initiation (No. 18-0051-S1A0), and the investigators complied with the protocol of IACUC. We purchased a total of 13 female pigs weighing 40–50 kg for this study, which were used to evaluate drug distribution (*n* = 4), tissue concentrations and penetration depth (*n* = 6), and pharmacokinetics and safety (*n* = 3) based on the types of intraperitoneal chemotherapy.

Before intraperitoneal chemotherapy, we applied capnoperitoneum by CO_2_ insufflation via a Veress needle to each pig, and then inserted two or three 12-mm bladeless trocars (Eagleport^®^; Dalim Medical Corp., Seoul, South Korea) along the midline of the abdomen, which was used as a passage for inserting DreamPen^®^ (Dalim Biotech, Gangwon, South Korea) and laparoscopic devices (STRIKER Korea CO., Ltd., Korea). After inserting the nozzle through the trocar directly down to the ileum, PIPAC and RIPAC were applied using 150 ml of 1% methylene blue to evaluate drug distribution and 3.5 mg of doxorubicin in 50 ml of 0.9% NaCl to evaluate pharmacokinetics, tissue concentrations, and toxicities.

### 2.4. Drug distribution

During PIPAC, 1% methylene blue solution was aerosolized via the nozzle with a velocity of 5 km/h at a flow rate of 30 ml/min under a pressure of 7 bars, whereas the nozzle was additionally rotated during spraying the solution as aerosol by RIPAC. After we completed the injection by PIPAC and RIPAC in each of two pigs, capnoperitoneum of 12 mmHg was maintained for 30 min (Supplementary Videos S1 and S2), and the pigs were euthanized. After that, the distribution and intensity of 1% methylene blue in the parietal and visceral peritoneum in the PIPAC and RIPAC pigs were compared with the naked eye. The three authors (GWY, SHS and SJC) evaluated the distribution and intensity without information on how to treat. Among them, the two authors (GWY and SHS) investigated them, and any discrepancies were addressed by a joint reevaluation with the third author (SJC).

### 2.5. Tissue concentration and penetration depth

We generated a modified Peritoneal Cancer Index (PCI) using the PCI for patients with PM (Jacquet & Sugarbaker, 1996). The modified PCI included nine parietal regions, including the central, right upper, epigastrium, left upper, left flank, left lower, pelvis, right lower, and right flank regions, and three visceral regions, which included the ileal, jejunal, and gastric regions (Supplementary Figure S1). After we sprayed 3.5 mg of doxorubicin in 50 ml of 0.9% NaCl by PIPAC and RIPAC in each of three pigs, we maintained capnoperitoneum of 12 mmHg for 30 min as mentioned above, and then, obtained two specimens of 2 × 2 cm-sized peritoneal tissue from each region of six pigs according to the modified PCI.

For tissue concentrations, all tissue specimens were stored at −80 °C and homogenized with a solvent consisting of a 1:1 mixture of methanol and 1% acetic acid equivalents to twice the weight of the tissue specimens. Then, the homogenized tissues were mixed with 1 ml of ethanol and vortexed for 30 min and held overnight in a refrigerator. After that, the mixture was centrifuged at 14,000 rpm for 10 min, and the supernatants were dried in a SpeedVac for 180 min at 45 °C. The samples were reconstituted to 50 µl, vortexed with 150 µl of acetonitrile with 50 mg/ml of daunorubicin as the internal standard for 30 s, and centrifuged at 13,000 rpm for 5 min. The supernatant (5 µl) was injected into HPLC for analysis.

To investigate the penetration depth of doxorubicin, we rinsed all tissue specimens with 0.9% NaCl solution to clean doxorubicin off the surface and then froze them in liquid nitrogen. We prepared cryosections with a thickness of 7 μm from three different specimen areas and applied DAPI. Thereafter, we estimated the depth of concentrated diffusion (DCD) and the depth of maximal diffusion (DMD) of doxorubicin in 12 regions by confocal laser scanning microscopy using a Leica TCS SP8 (Leica Mikrosysteme GmbH, Hessen, Germany) and compared them between the PIPAC and RIPAC treatments. In this study, we defined DCD as the distance between the luminal surface and the surface where positive doxorubicin staining was most accumulated, and DMD as the distance between the luminal surface and the innermost depth at which positive doxorubicin staining was visualized. The three authors (SP, WL and GS) investigated DCD and DMD without information on how to treat. Among them, the two authors (SP and WL) investigated them, and inconsistencies were resolved through joint reevaluation with the third author (GS).

### 2.6. Pharmacokinetics and toxicities

For evaluating the pharmacokinetics of RIPAC using doxorubicin, we collected blood from three pigs a total of 11 times as follows: before RIPAC, after 15 min, after 30 min, after 45 min, after 1 h, after 1.25 h, after 1.5 h, after 1.75 h, after 2 h, after 24 h, and after 48 h. Then, 50 µl of serum and 100 µl of 0.1% formic acid acetonitrile with 15 mg/ml of daunorubicin as the internal standard were vortexed for 30 min. The mixtures were centrifuged at 14,000 rpm for 10 min, and 5 µl of the supernatants were injected into HPLC for analysis.

To investigate renal and hepatic toxicities, we collected blood from three pigs a total of six times as follows: before RIPAC, immediately after RIPAC, and after one to four days. Aspartate aminotransferase (AST), alanine aminotransferase (ALT), gamma-glutamyl transpeptidase (GGT), bilirubin, alkaline phosphatase (ALP), creatinine, and C-reactive protein (CRP) were measured in the serum.

### 2.7. Liquid chromatography and tandem mass spectrometry

We analyzed the serum and tissue concentrations of doxorubicin by high-performance liquid chromatography (HPLC) using an Agilent 1260 Infinity (Agilent, Santa Clara, CA, USA), followed by tandem mass spectrometry (MS/MS) using API4000QTRAP (Applied Biosystems, Waltham, MA, USA). For the HPLC analysis, a Gemini 5 μm C18, 50 × 2.0 mm analytical column (Phenomenex, Torrance, CA, USA) was used. The mobile phase consisted of 5 mM ammonium acetate and 0.1% acetic acid acetonitrile with a flow rate of 0.3 ml/min and a 25 °C column temperature over 7.5 min.

The MS/MS was equipped with a positive ionization mode with Turbo Spray, and multiple reaction monitoring was used for quantification. The nebulizer and desolvation gas pressure was 50 psi, both using nitrogen. MS/MS was conducted under a needle voltage of 5000 V and a set temperature of 400 °C. The acquisition delay was 0 s with a pause time of 5 ms.

### 2.8. Statistical analysis

A null hypothesis was an assumption that there were no differences in the homogeneity of spatial distribution, tissue concentrations, and penetration depth of agents between PIPAC and RIPAC. For testing the null hypothesis, the continuous variables were analyzed by the Kruskal-Wallis test, and the Mann-Whitney *U* test in SPSS version 22 software (IBM Corp., Armonk, NY, USA, RRID:SCR_002865), and *p* ≤ .05 was considered significant because of the non-parametric tests.

Moreover, we performed a pharmacokinetic study for RIPAC with doxorubicin, based on a non-compartmental model using R software for pharmacokinetic analysis. To characterize the pharmacokinetic analysis, the peak serum concentration (*C*_max,_ mg/ml) and the time to the peak serum concentration (*T*_max_, hour) were identified. Then, the area under the curve (AUC, mg/ml × hour) of the individual pharmacokinetic curve was calculated using the linear trapezoidal rule from zero to the time of the last observed positive concentration.

## 3. Results

### 3.1. Drug distribution

**Figure 2.**
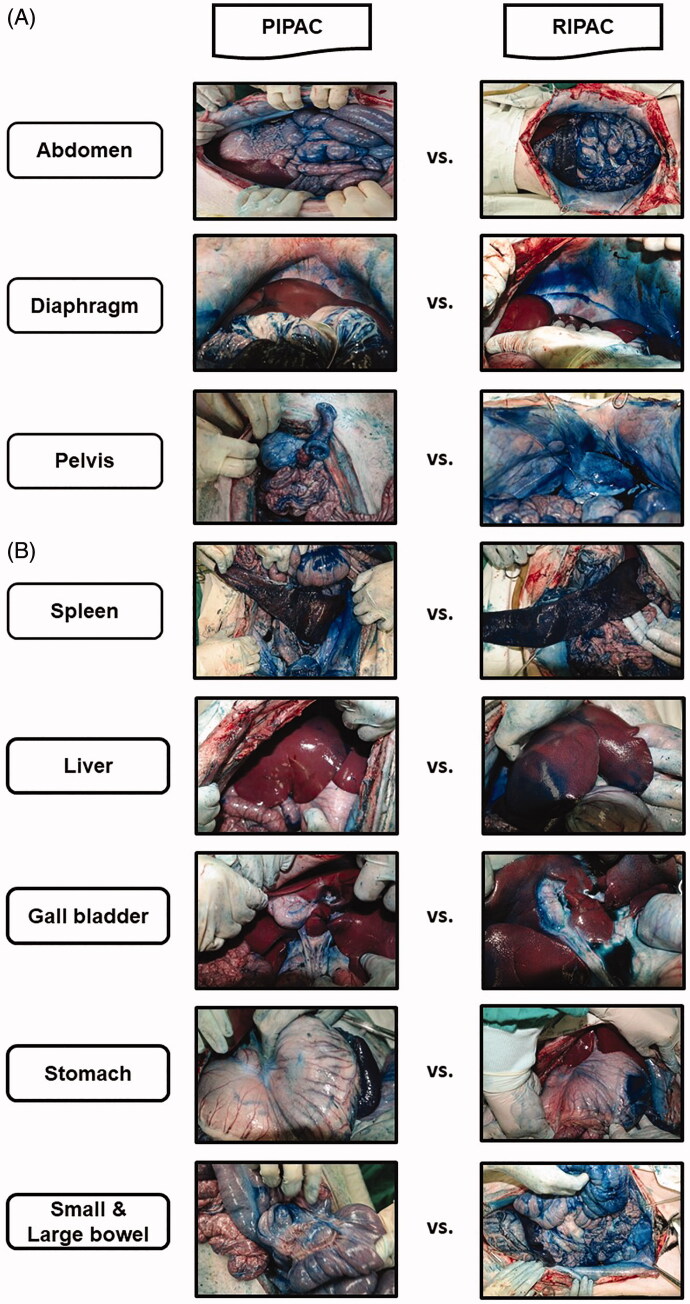
Comparison of the distribution and intensity of 1% methylene blue staining in pressurized intraperitoneal aerosol chemotherapy (PIPAC), and rotational intraperitoneal pressurized aerosol chemotherapy (RIPAC) in (A) the parietal and (B) visceral peritoneum.

When we compared the distribution and intensity of 1% methylene blue staining between PIPAC and RIPAC in the parietal peritoneum, the distribution was wider, and the intensity was stronger after RIPAC than after PIPAC. Especially, 1% methylene blue staining was observed in gravity-dependent regions after PIPAC, whereas all regions, regardless of gravity, were also strongly stained after RIPAC. Furthermore, the distribution was also the most comprehensive, and the intensity was also the most vigorous after RIPAC in the visceral peritoneum of the spleen, liver, gall bladder, stomach, small and large bowels, and mesentery (Figure 2).

### 3.2. Tissue concentrations and penetration depth

**Figure 3.**
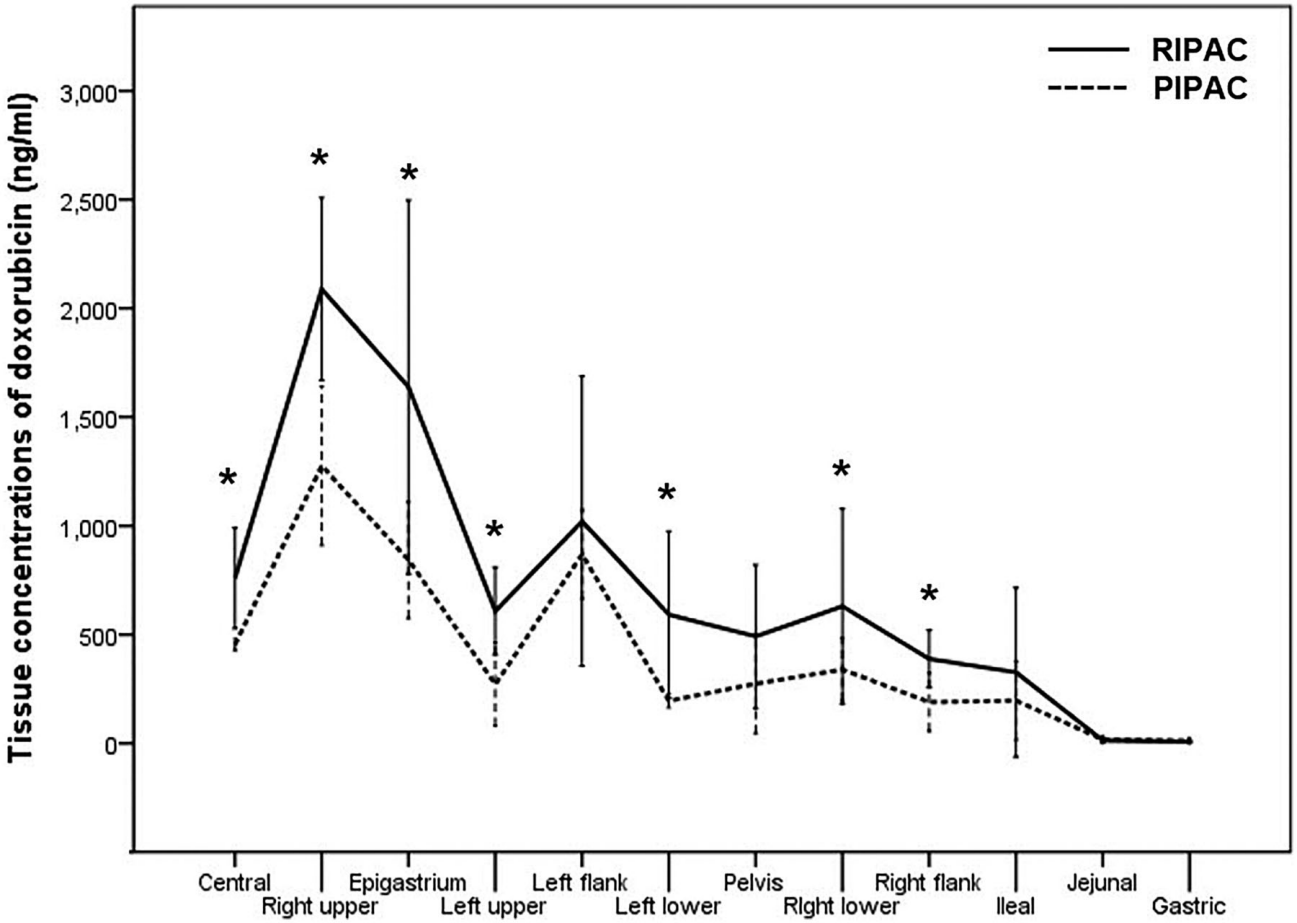
Comparison of tissue concentrations of doxorubicin between pressurized intraperitoneal aerosol chemotherapy (PIPAC) and rotational intraperitoneal pressurized aerosol chemotherapy (RIPAC) according to the modified Peritoneal Cancer Index (*p ≤ .05).

When we compared the tissue concentrations of doxorubicin between PIPAC and RIPAC according to the modified PCI, there were no differences in the tissue concentrations of doxorubicin in the left flank, pelvis, ileal, jejunal, and gastric regions, whereas the tissue concentrations of doxorubicin were higher after RIPAC than after PIPAC in the central, right upper, epigastrium, left upper, left lower, right lower, and right flank regions (Figure 3 and Supplementary Table S1).

**Figure 4.**
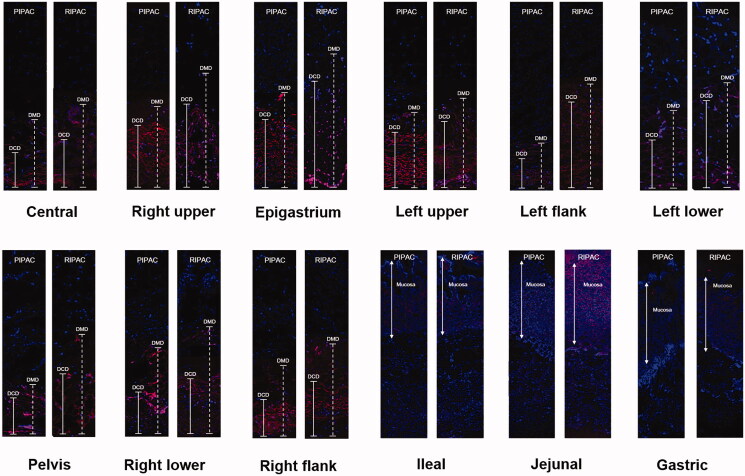
The depth of concentrated diffusion (DCD) and the depth of maximal diffusion (DMD) using confocal laser scanning microscopy in pressurized intraperitoneal aerosol chemotherapy (PIPAC) and rotational intraperitoneal pressurized aerosol chemotherapy (RIPAC) according to the modified Peritoneal Cancer Index.

**Figure 5.**
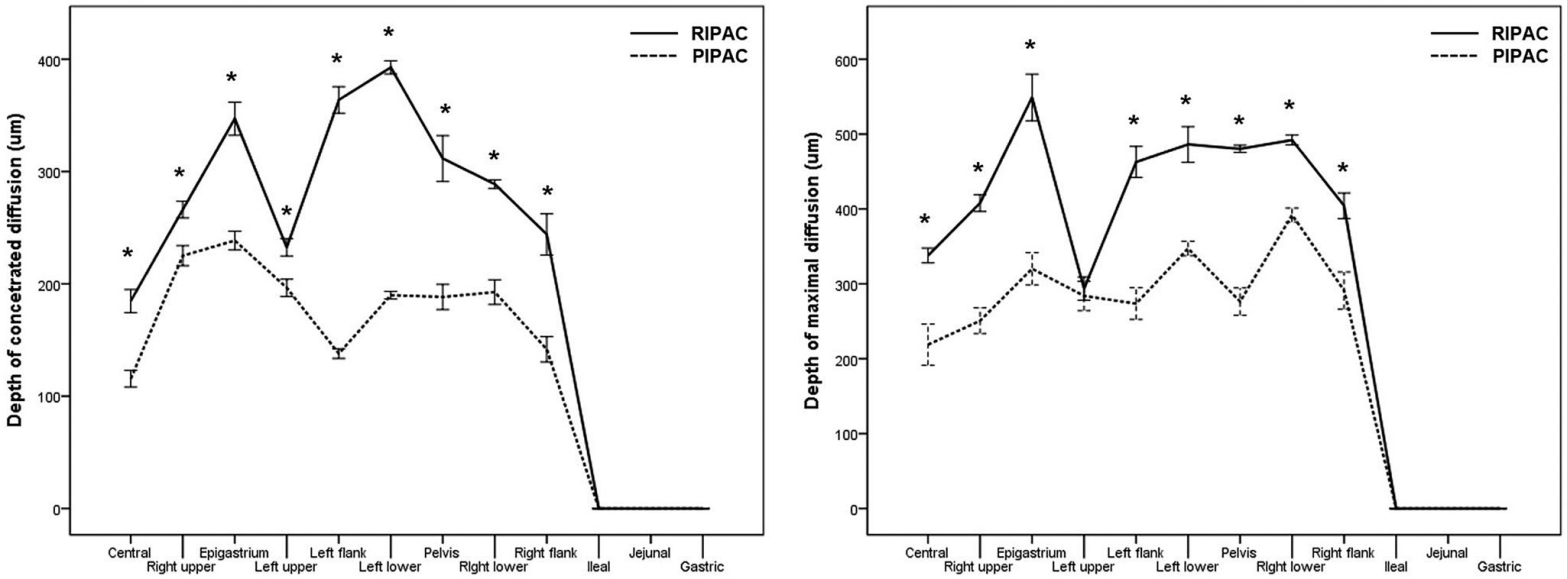
Comparison of the depth of concentrated diffusion (DCD) and the depth of maximal diffusion (DMD) between pressurized intraperitoneal aerosol chemotherapy (PIPAC) and rotational intraperitoneal pressurized aerosol chemotherapy (RIPAC) according to the modified Peritoneal Cancer Index (*p ≤ .05).

Figure 4 depicts the comparison of the penetration depth of doxorubicin between PIPAC and RIPAC. In terms of the penetration depth of doxorubicin, the DCD was higher after RIPAC than after PIPAC in the central, right upper, epigastrium, left upper, left flank, left lower, pelvis, right lower, and right flank regions. DMD was also higher after RIPAC than after PIPAC in the central, right upper, epigastrium, left flank, left lower, pelvis, right lower, and right flank regions. However, doxorubicin did not penetrate the peritoneum of the ileal, jejunal, and gastric areas in either the PIPAC or RIPAC pigs. Only mucosal doxorubicin staining without penetration into the peritoneum was observed (Figure 5 and Supplementary Table S2).

### 3.3. Pharmacokinetics and toxicities

**Figure 6.**
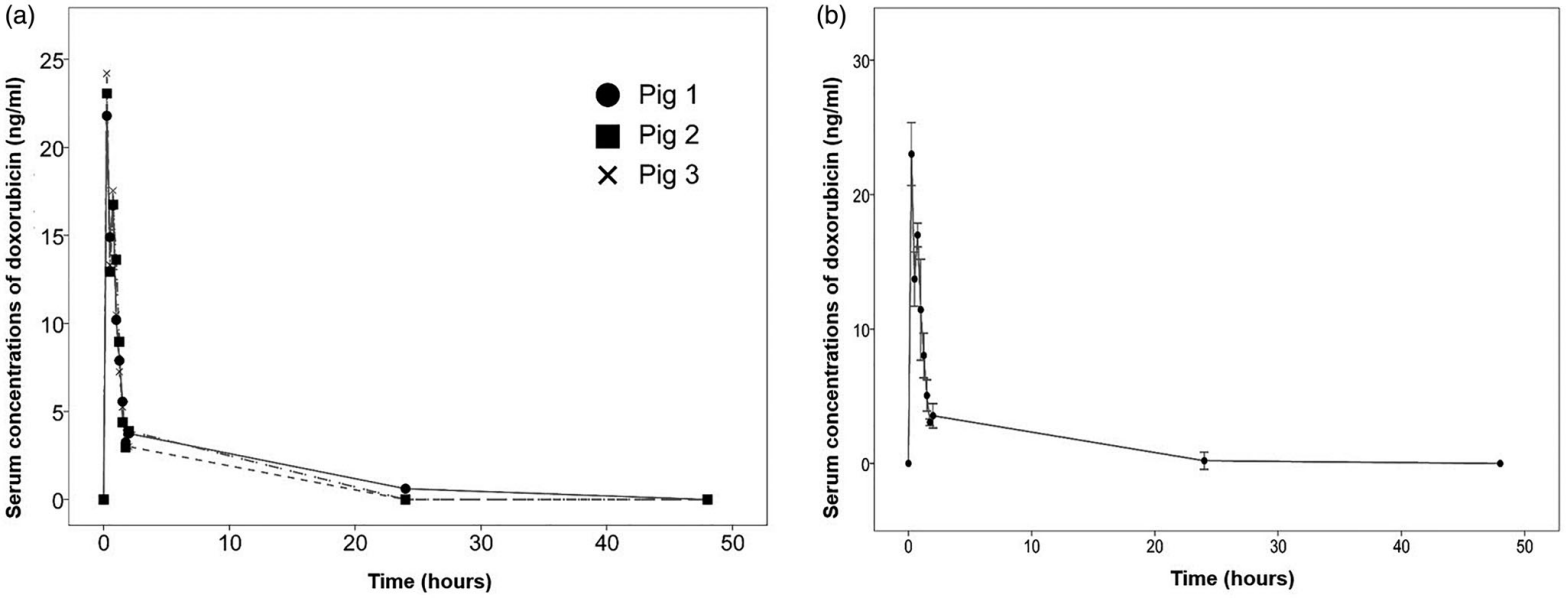
The pharmacokinetic properties of doxorubicin after rotational intraperitoneal pressurized aerosol chemotherapy (RIPAC). (A) Individual data and (B) group data.

The time-dependent serum concentrations and pharmacokinetic properties of doxorubicin used in RIPAC are depicted in Supplementary Table S3. The mean values of *C*_max_, AUC, and *T*_max_ were 23.02 mg/ml, 20.9 mg/ml × h, and 0.25 h, respectively. All pigs showed a similar pharmacokinetic property pattern, in which the serum concentrations of doxorubicin reached a peak after 15 min, decreased after 30 min, increased again after 45 min, and decreased over 48 h (Figure 6). Table 1 shows the renal and hepatic toxicities before and after RIPAC with doxorubicin. The results showed no differences in creatinine, bilirubin, ALP, AST, ALT, GGT, or CRP before RIPAC, immediately after RIPAC, or on days 1, 2, 3, or 4.

**Table 1.** Comparison of toxicities related to rotational intraperitoneal pressurized aerosol chemotherapy (RIPAC) with doxorubicin.

Parameters	Measurement time							
	Before RIPAC	Immediately after RIPAC	Day 1	Day 2	Day 3	Day 4	Day 5	*p* Value
Creatinine (mg/dl)	0.99 (0.63, 1.17)	1.01 (0.68, 1.35)	0.82 (0.77, 1.17)	0.78 (0.73, 1.07)	0.84 (0.7, 1.03)	0.85 (0.66, 1.07)	0.78 (0.78, 1.16)	1.00
Bilirubin (mg/dl)	0.09 (0.02, 0.15)	0.03 (0.01, 0.15)	0.05 (0.04, 0.15)	0.04 (0.02, 0.15)	0.04 (0.02, 0.15)	0.04 (0.02, 0.15)	0.06 (0.03, 0.15)	.97
ALP (IU/l)	88.4 (58.9, 95)	77.1 (57, 88)	68.6 (52.1, 71.3)	69.6 (55.1, 83.3)	69.6 (51.3, 96.7)	70.8 (53.1, 90.7)	67.1 (51.4, 82.3)	.98
AST (IU/l)	60 (21, 69)	50 (38, 64,3)	83 (31, 98)	95 (36, 122)	69 (20, 154)	86 (21, 97.1)	83 (38, 92.8)	.98
ALT (IU/l)	37 (24, 55)	37 (23, 54)	38 (24, 38)	45 (22, 50)	51 (21, 55)	50 (21, 68)	63 (20, 64)	.98
GGT (IU/l)	59 (21, 76)	49 (22, 63)	53 (26, 56)	48 (27, 59)	54 (25, 56)	51 (24, 64)	55 (25, 90)	1.00
CRP (g/l)	0.01 (0, 0.01)	0 (0, 0.1)	0 (0, 0.1)	0.01 (0.01, 0.1)	0.01 (0.01, 0.1)	0.01 (0, 0.1)	0 (0, 0.1)	.89

ALP: alkaline phosphatase; ALT: alanine aminotransferase; AST: aspartate aminotransferase; CRP: C-reactive protein; GGT: gamma-glutamyl transferase.

All values were shown as median with range.

## 4. Discussion

PIPAC has been suggested to be useful as palliative therapy for PM of recurrent or refractory solid tumors, which may lead to histologic regression, and thereby improve the quality of life (Tempfer et al., 2015; Horvath et al., 2018; Alyami et al., 2020; Ellebaek et al., 2020). Even if chemotherapeutic agents shown to be resistant in intravenous chemotherapy are used again in PIPAC, the agents may be absorbed into the peritoneal tumors by passive diffusion, which can be effective for treating PM by maintaining higher concentrations within tumor tissues while minimizing systemic absorption (Yan et al., 2010; Alyami et al., 2019). However, compartmentalization by inadequate exposure of the entire peritoneal cavity due to postoperative adhesion, individual differences in the three-dimensional structure of the peritoneal cavity, and aerosol delivery capacity limited by gravity can hinder homogeneous distribution and effective penetration of chemotherapeutic agents into the peritoneum during PIPAC. For enhancing drug delivery during PIPAC, the nozzle rotation can change the spray direction, thereby improving the homogeneous distribution of chemotherapeutic agents (Khosrawipour et al., 2016a). Thus, this study tried to provide preclinical evidence showing that RIPAC developed by the KoRIA trial group may enhance drug delivery compared to PIPAC with fewer toxicities.

First of all, RIPAC was superior to PIPAC in terms of the distribution and intensity of the chemotherapeutic agents. Even though aerosols with a median diameter of 25 μm are injected into the peritoneal cavity with a velocity of 60 km/h through CapnoPen^®^ (Capnomed, Villingendorf, Germany) (Khosrawipour et al., 2016c), our prototype shows that droplets with a median diameter of 30 μm are sprayed with a velocity of 5 km/h through DreamPen^®^ (Dalim Biotech, Gangwon, South Korea) under the same flow rate of 30 ml/min (Lee et al., 2020). This means that the nozzle injection outlet may be larger in DreamPen^®^ (Dalim Biotech, Gangwon, South Korea) than in CapnoPen^®^ (Capnomed, Villingendorf, Germany), which can reduce the turbulent flow of aerosols (Klabunde, 2012). Subsequently, most of aerosols may move through DreamPen^®^ (Dalim Biotech, Gangwon, South Korea) according to the inertia created by the injection pressure, and more collision may allow aerosols to move to various regions of the peritoneum by longer breakup-length within the sprayed zone, which may lead to the increased movement of aerosols by an increase of subsequent deflection (Flagan and Seinfeld, 1988; Yoon et al., 2004; Piao et al., 2021). This hypothesis can be supported by the different finding that the penetration depth of doxorubicin was minimal (20–150 μm) in the other regions except for the opposite side of CapnoPen^®^ (Capnomed, Villingendorf, Germany) (Khosrawipour et al., 2016a, 2016b, 2016c), whereas it ranged from 220 μm to 480 μm after spraying aerosol by DreamPen^®^ (Dalim Biotech, Gangwon, South Korea) in most of regions of the peritoneum (Piao et al., 2021).

Additionally, the enhancement of drug delivery by RIPAC improved the tissue concentrations and penetration depth of doxorubicin despite the lower injection pressure of aerosols in RIPAC compared to the conventional PIPAC (101 vs. 200 psi). These findings mean that more droplets of doxorubicin after RIPAC may reach various regions in the peritoneal cavity. A previous *ex vivo* study supported these findings, where the penetration depth increased as the doxorubicin concentration increased under the same condition of capnoperitoneum of 12 mmHg for 30 min (Khosrawipour et al., 2016b).

In particular, it is important that the DCD was higher in RIPAC than in PIPAC because the area with a concentrated population of cells affected by doxorubicin can promote cytotoxic effects (Lee et al., 2020). Moreover, the DMD after PIPAC in this study was similar to the value reported in a previous study (≤400 μm) (Khosrawipour et al., 2016c), and RIPAC showed a higher DMD than PIPAC (≤500 μm) in most of the regions in the peritoneal cavity, indicating that RIPAC may be more advantageous for the passive diffusion of chemotherapeutic agents than PIPAC.

In terms of the pharmacokinetic properties of doxorubicin used in RIPAC, the serum concentrations peaked after 15 min, decreased after 30 min, increased again after 45 min, and decreased over 48 h. These pharmacokinetic properties of doxorubicin were different from those reported in a previous study where the serum concentrations of doxorubicin decreased after the peak was reached 30 min after PIPAC (Solass et al., 2014). However, the pharmacokinetic changes in doxorubicin in this study seem to make more sense when we consider hemodynamic changes during laparoscopic surgery. During laparoscopic surgery, the compression of the inferior vena cava and the portal vein may lead to a decrease in venous return and an increase in systemic vascular resistance, which could decrease the systemic absorption of doxorubicin. However, subsequent changes in the endocrine system may decrease systemic vascular resistance, which could increase the systemic absorption of doxorubicin again (Chui et al., 1993). A previous study reported that serum levels of vasopressin may reduce 30 min after capnoperitoneum, and thereby, systemic vascular resistance may decrease and cardiac output may increase, supporting the pharmacokinetic properties of doxorubicin after RIPAC in this study (Joris et al., 1998). Furthermore, there were no renal and hepatic toxicities caused by RIPAC with doxorubicin consistent with relevant studies that showed a similar safety profile after PIPAC (Blanco et al., 2013; Solaß et al., 2013; Ametsbichler et al., 2018). The results indicate that RIPAC can be safely conducted like PIPAC in a clinical setting.

In contrast, we found no penetration of doxorubicin into the peritoneum of the ileal, jejunal, and gastric regions in either RIPAC or PIPAC. Although the penetration depth was highest in the small bowel located directly under the nozzle in a previous study (Khosrawipour et al., 2016c), we found no penetration of doxorubicin into the peritoneum of the ileal region despite being located directly under the nozzle. Although the relevant evidence is not definitive, we hypothesized that differences in the histologic structures between the visceral and parietal peritoneum rather than the position of the nozzle could lead to the concentration distributions. When we consider that the penetration depth of RIPAC may range within 500 µm, we can expect that doxorubicin can penetrate soft extraperitoneal fat tissues beyond the parietal peritoneum (Abrahams et al., 2019), whereas penetration into the dense muscularis layer beyond the visceral peritoneum seems difficult (van Baal et al., 2017; Isaza-Restrepo et al., 2018). Our findings that the tissue concentrations of doxorubicin were lower in the visceral peritoneum than in the parietal peritoneum support this hypothesis, and lower tissue concentrations in the visceral peritoneum seemed to be related to the systemic absorption of doxorubicin in the mucosal layer instead of the direct penetration of doxorubicin into the peritoneum.

Nevertheless, this study has some limitations as follows. First, these results should be validated on biomedical engineering evidence that RIPAC may be more advantageous than PIPAC. Second, the results from this study were exploratory due to a small number of pigs and a subsequent lack of statistical power, which should be proved in large-scale preclinical studies. Third, we focused on the penetration depth of doxorubicin in the visceral peritoneum of small and large bowels among the internal organs and did not evaluate it in various types of organs including the liver and spleen because most of patients with PM showed malignant bowel obstruction during disease progression, and PIPAC is needed to improve quality of life related to bowel function (Tempfer et al., 2015; Alyami et al., 2019). Fourth, whether improved drug delivery and penetration into the peritoneum by RIPAC may be more effective for treating PM should be proven in clinical trials.

## 5. Conclusions

The KoRIA trial group developed RIPAC to enhance drug delivery into peritoneal tumors and reported preclinical evidence that RIPAC may improve drug distribution, tissue concentrations, and penetration depth compared to PIPAC. Moreover, the pharmacokinetic properties after RIPAC may be determined by hemodynamic changes during capnoperitoneum, and renal and hepatic toxicities were not seen in RIPAC, suggesting that RIPAC can be effectively and safely introduced into clinical settings.

## Supplementary Material

Supplemental Material

## Patent

SJP, EJL, HSL, JK, JCL, and HSK have a Korean pending patent and Patent Cooperation Treaty entitled “Rotational Injection of Pressurized System for Intraperitoneal Chemotherapy” (KR1020200011994; PCT/KR2021/000573)
