# Supplementary material for: Development of rotational intraperitoneal pressurized aerosol chemotherapy to enhance drug delivery into the peritoneum
Source: Drug Deliv. 2021 Jun 12;28(1):1179–87. doi: 10.1080/10717544.2021.1937382 (PMC8204987; doi:10.1080/10717544.2021.1937382)
Supplement: Supplemental Material [file IDRD_A_1937382_SM1350.zip › Supplementary Table S1.docx]

Supplementary Table S1. Comparison of tissue concentrations of doxorubicin between pressurized intraperitoneal aerosol chemotherapy (PIPAC) and rotational injection of pressurized intraperitoneal aerosol chemotherapy (RIPAC)

|  | PIPAC | RIPAC | P value |
| --- | --- | --- | --- |
| Central (ng/ml) | 448.1 (445.5, 469) | 729 (663.1, 888.3) | 0.05 |
| Right upper (ng/ml) | 1240 (1113.9, 1473.6) | 2124.8 (1862, 2277) | 0.05 |
| Epigastrium (ng/ml) | 819.6 (723.6, 988.7) | 1860.9 (1143, 1910.5) | 0.05 |
| Left upper (ng/ml) | 217.3 (214.3, 381.8) | 643.7 (496, 687.4) | 0.05 |
| Left flank (ng/ml) | 912.5 (752.8, 944) | 916.2 (753.5, 1394) | 0.51 |
| Left lower (ng/ml) | 189.6 (183.4, 213) | 651.6 (378.2, 747) | 0.05 |
| Pelvis (ng/ml) | 213 (203.5, 406.5) | 434 (364, 678) | 0.13 |
| Right lower (ng/ml) | 341.4 (266.1, 411.5) | 541 (465, 885.9) | 0.05 |
| Right flank (ng/ml) | 161.7 (141.4, 266.1) | 373.5 (331, 460.8) | 0.05 |
| Ileal (ng/ml) | 187 (111.4, 291.5) | 216.7 (212.6, 551.1) | 0.28 |
| Jejunal (ng/ml) | 21.2 (8.1, 24.4) | 16.5 (8.6, 16.6) | 0.51 |
| Gastric (ng/ml) | 9.3 (7.3, 19) | 6.4 (5.9, 10.3) | 0.28 |

All values were shown as median and range (ng/ml).
