# Supplementary material for: Development of rotational intraperitoneal pressurized aerosol chemotherapy to enhance drug delivery into the peritoneum
Source: Drug Deliv. 2021 Jun 12;28(1):1179–87. doi: 10.1080/10717544.2021.1937382 (PMC8204987; doi:10.1080/10717544.2021.1937382)
Supplement: Supplemental Material [file IDRD_A_1937382_SM1350.zip › Supplementary Table S2.docx]

Supplementary Table S2. Comparison of the depth of concentrated diffusion (DCD) and depth of maximal diffusion (DMD) of doxorubicin between pressurized intraperitoneal aerosol chemotherapy (PIPAC) and rotational injection of pressurized intraperitoneal aerosol chemotherapy (RIPAC) according to the modified Peritoneal Cancer Index

| Properties | Depth of concentrated diffusion | | | Depth of maximal diffusion | | |
| --- | --- | --- | --- | --- | --- | --- |
|  | PIPAC | RIPAC | P value | PIPAC | RIPAC | P value |
| Central (μm) | 116.9 (111.3, 118.3) | 183.7 (180.1, 190.2) | 0.05 | 224.1 (203.2, 229.1) | 338.4 (332.5, 342.3) | 0.05 |
| Right upper (μm) | 223.2 (221.8, 230.1) | 267.4 (261.9, 269) | 0.05 | 250.5 (242.3, 259.4) | 409.1 (401.8, 412.9) | 0.05 |
| Epigastrium (μm) | 240.1 (233.9, 241.7) | 349.6 (338.7, 352.7) | 0.05 | 322.1 (308.6, 329.7) | 551.2 (532.4, 563.2) | 0.05 |
| Left upper (μm) | 196.3 (192.6, 200.3) | 232.5 (228.6, 236.4) | 0.05 | 280.3 (276.3, 295) | 291.2 (287.3, 302.3) | 0.28 |
| Left flank (μm) | 137.6 (135.9, 140.2) | 274.9 (262.4, 283.4) | 0.05 | 362.4 (358.6, 370.2) | 467.3 (450.8, 470.1) | 0.05 |
| Left lower (μm) | 190.7 (188, 191) | 392.7 (389.7, 395.6) | 0.05 | 345.9 (343.1, 352.5) | 482.9 (476.3, 499.4) | 0.05 |
| Pelvis (μm) | 190.3 (182, 192.7) | 313.2 (300.7, 320.9) | 0.05 | 278.9 (266.2, 283.8) | 480.8 (477.7, 482.3) | 0.05 |
| Right lower (μm) | 193.4 (186.8, 197.7) | 289.3 (286.7, 290.3) | 0.05 | 390.7 (387.9, 397) | 390.6 (489.7, 495.8) | 0.05 |
| Right flank (μm) | 140.2 (136.9, 147.9) | 243.8 (234.9, 253.2) | 0.05 | 289.4 (279.3, 304) | 403.2 (396.2, 413.2) | 0.05 |
| Ileal (μm) | 0 | 0 | 1.00 | 0 | 0 | 1.00 |
| Jejunal (μm) | 0 | 0 | 1.00 | 0 | 0 | 1.00 |
| Gastric (μm) | 0 | 0 | 1.00 | 0 | 0 | 1.00 |

All values were shown as median and range (μm).
