# Supplementary material for: Development of rotational intraperitoneal pressurized aerosol chemotherapy to enhance drug delivery into the peritoneum
Source: Drug Deliv. 2021 Jun 12;28(1):1179–87. doi: 10.1080/10717544.2021.1937382 (PMC8204987; doi:10.1080/10717544.2021.1937382)
Supplement: Supplemental Material [file IDRD_A_1937382_SM1350.zip › Supplementary Table S3.docx]

Supplementary Table S3. Time-dependent serum concentrations and pharmacokinetic properties of doxorubicin used in rotational intraperitoneal pressurized aerosol chemotherapy (RIPAC)

| Time | RIPAC |
| --- | --- |
| Before RIPAC (median, range, ng/ml) | 0 (0, 0) |
| After 15 minutes (median, range, ng/ml) | 23.06 (21.8, 24.2) |
| After 30 minutes (median, range, ng/ml) | 13.31 (12.94, 14.9) |
| After 45 minutes (median, range, ng/ml) | 16.75 (16.7, 17.54) |
| After 1 hr (median, range, ng/ml) | 10.48 (10.2, 13.63) |
| After 1.25 hr (median, range, ng/ml) | 7.89 (7.26, 8.96) |
| After 1.5 hr (median, range, ng/ml) | 5.25 (4.38, 5.56) |
| After 1.75 hr (median, range, ng/ml) | 3 (2.95, 3.24) |
| After 2 hr (median, range, ng/ml) | 3.75 (3, 3.9) |
| After 24 hours (median, range, ng/ml) | 0 (0, 0.61) |
| After 48 hours (median, range, ng/ml) | 0 (0, 0) |
| C_max_ (mean, SD, CV, ng/ml) | 23.02, 1.2, 0.05 |
| AUC* (mean, SD, CV, ng/ml×hr) | 20.9, 21.81, 0.66 |
| T_max_ (mean, SD, CV, hr) | 0.25, 0, 0 |

Abbreviations: AUC, the area under the curve; C_max_, the peak serum concentration; CV, coefficient of variation; SD, standard deviation; T_max_, the time measurement to C_max_.

^*^AUC was calculated from the time zero to the time of the last positive concentrations.
