# Supplementary figures and images for: Development of rotational intraperitoneal pressurized aerosol chemotherapy to enhance drug delivery into the peritoneum
Source: Drug Deliv. 2021 Jun 12;28(1):1179–87. doi: 10.1080/10717544.2021.1937382 (PMC8204987; doi:10.1080/10717544.2021.1937382)

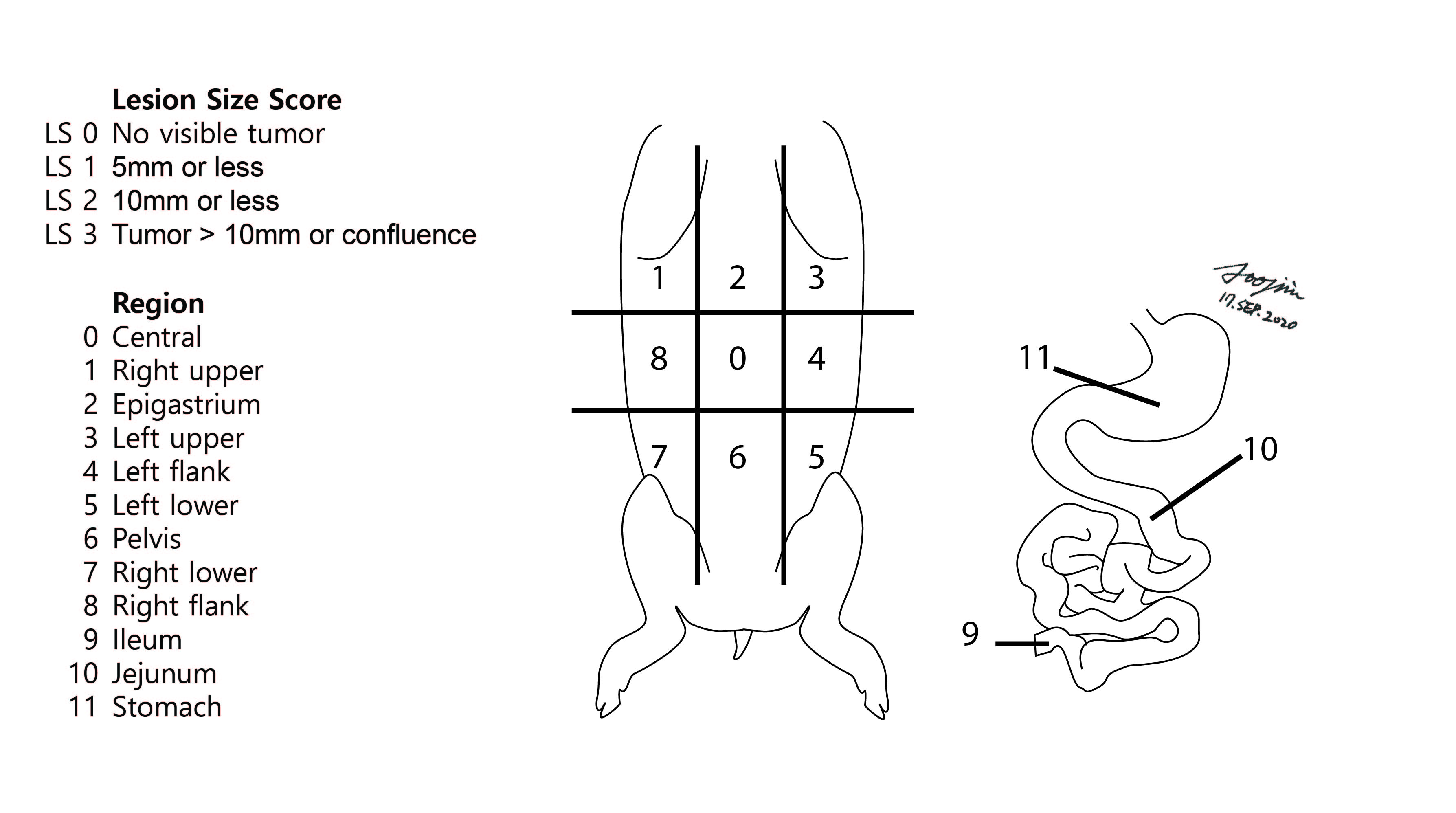

Supplement: Supplemental Material [file IDRD_A_1937382_SM1350.zip › Supplementary Figure S1.jpg]
